# Advancement in Scaffold-Based 3D Cell Culture Models for Osteosarcoma Drug Screening

**DOI:** 10.1021/acsbiomaterials.5c01174

**Published:** 2025-10-27

**Authors:** Ponnamma Mandeda Madaiah, Rudra Nath Ghosh, Pramod K Namboothiri, Mathew Peter

**Affiliations:** † Manipal School of Life Sciences, 539679 Manipal Academy of Higher Education, Manipal 576104, India; ‡ Department of Biomedical Engineering, Manipal Institute of Technology, 76793 Manipal Academy of Higher Education, Manipal 576104, India

**Keywords:** tumor microenvironment, drug resistance, extracellular matrix, biomimetic scaffolds, chemoresistance

## Abstract

Osteosarcoma (OS), an extremely aggressive bone cancer that primarily occurs in children and teenagers, continues to pose critical clinical challenges due to its high propensity for metastasis, resistance to conventional therapies, and lack of specific biomarkers for early detection. Despite advances in surgical techniques and chemotherapeutic regimens, patient outcomes remain suboptimal, predominantly because conventional two-dimensional (2D) cell culture systems do not accurately mimic the intricate tumor microenvironment (TME), which often results in limited success when translating preclinical results to clinical success. In response to the shortcomings, the field has shifted toward three-dimensional (3D) culture systems, which more accurately mimic the spatial, mechanical, and biochemical characteristics of native OS TME. This review systematically examines the evolution and current state of 3D OS models, with a particular focus on scaffold-based systems. These models, utilizing biomimetic scaffolds provide enhanced platforms for studying tumor–stroma interactions, drug responses, and chemoresistance. It also briefs the use of scaffold-free spheroid models, which, despite their utility in replicating certain aspects of tumor heterogeneity and cell–cell interactions, are limited in their ability to fully emulate the *in vivo* microenvironment. The review further discusses technical and translational hurdles, such as optimizing scaffold properties and integrating patient-derived cells, which must be addressed to realize the full potential of 3D models in personalized medicine and drug discovery. The significant advancement of scaffold-based 3D OS models offers a more physiologically relevant platforms to bridge the gap between experimental research and clinical application in chemotherapy.

## Graphical abstract

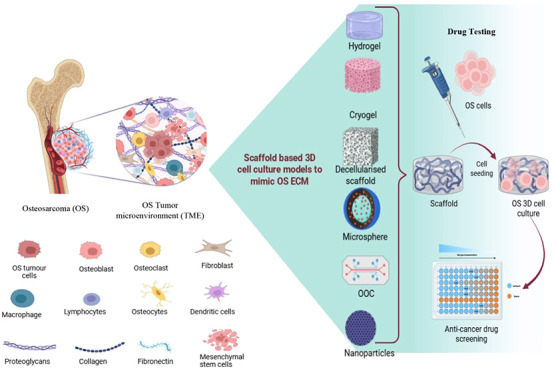

## 1 Introduction

Osteosarcoma (OS) is one of the most widely prevalent aggressive bone malignancies, predominantly found in children and adolescents.^1^ Despite decades of research and incremental improvements in multimodal therapy, including surgery and combination chemotherapy, the prognosis for patients with OS remains challenging. A significant portion of OS patients are still resistant to standard-of-care therapy, and the mortality rate remains alarmingly high.^2^ There are two major reasons for the high mortality rate associated with OS: First, OS metastasizes to major organs such as lungs which increases the chances of comorbidity and mortality.^3^ Second, these cancer cells do not have specific markers, making early detection difficult.^4^

Although there have been significant advancements in chemotherapy and surgical treatments for osteosarcoma (OS) over the past few decades, many patients still develop resistance to therapy. This resistance often results in the need for surgical resection of the affected bone. Even after such interventions, patients are at risk for disease recurrence, which can ultimately progress to metastatic disease. It is common for clinicians and patients to experience great distress due to difficulties in OS treatment, recurrence, and metastasis.^5^ The development of novel therapeutic approaches and the establishment of more predictive preclinical models are top priorities to facilitate the efficient translation of experimental findings into successful clinical treatments.^6^ The invention of novel treatments for OS is hindered by the lack of *in vitro* models that accurately replicate the tissue architecture, molecular markers, and drug responses seen in OS patients.

Although conventional two-dimensional (2D) cell culture systems have long served as the foundation for OS research and drug screening, they do not accurately epitomize the complex pharmacological and physiological responses at the organ level. 2D cultures fail to accurately mimic the complexity of the *in vivo* tumor microenvironment (TME) for drug testing. This shortcoming is due to the lack of cell–cell and cell–matrix interactions and the absence of dynamic microenvironmental cues that characterize human bone tumors *in vivo,* which leads to limited clinical translation of therapeutic findings.^6,7^ The disparity between 2D cell cultures and more complex biological systems is the cause of significant failure rates of potential drugs in clinical trials. Compounds that appear effective in 2D cell culture conditions frequently struggle to produce the same results in animal models or human patients, contributing to high attrition rates in drug development due to their low *in vitro*-to-*in vivo* translational ability.^8^ As a result, there has been a significant transition in the field toward the creation and utilization of three-dimensional (3D) culture models that more accurately replicate the architectural, mechanical, and biochemical characteristics of OS tissue.^6^ In contrast to 2D cultured cells, 3D cell culture models mimic the spatial complexity of *in vivo* TME and have the ability to reproduce the physiological characteristics and function of the tumor with a greater level of accuracy. Moreover, when cancer cells are cultured in 3D for prolonged periods, due to their self-renewing and proliferating nature, they remain genetically stable without causing any mutation patterns.^9^ Therefore, 3D cell culture models are more relevant platforms to replicate the structural and functional intricacies of *in vivo* tissues, and to investigate the intricate dynamic processes such as tumor development.^10^ The transition to 3D cell culture represents a major advancement, offering a more physiologically relevant model for offering crucial insights into cancer research and drug development. Although 3D cell culture has some challenges, its advantages over traditional 2D culture make it a more effective and physiologically relevant model.^10^

3D cell culture models are usually categorized into scaffold-based systems and scaffold-free approaches. Although scaffold-free spheroid models are considered the gold standard among 3D culture systems, their limited ability to replicate complex cell–ECM interactions in the TME makes them impotent for studying OS prognosis and treatment. A significant amount of attention has been drawn to scaffold-based 3D culture systems for their ability to physically reinforce cell growth in a spatially organized manner, facilitate extracellular matrix (ECM) deposition, and study tumor–stroma interactions, ultimately enhancing cell survival and function.^11–15^ Biomimetic scaffolds composed of natural or synthetic materials can be used as models that provide a versatile platform for investigating OS biology and evaluating therapeutic responses. Therefore, scaffold-based 3D cell culture has become a critical tool in cancer research, offering a more physiologically accurate setting for examining tumor behavior, drug responses, and the interactions between cancer cells with the surrounding TME.^16^

This review article provides a comprehensive overview of the developments and current advancements in scaffold-based 3D OS models. It explores their effectiveness in replicating the tumor microenvironment, along with their contribution to identify clinically relevant drug responses, and analyzes their potential in advancing personalized medicine. Additionally, it addresses the technical and translational challenges that must be overcome for these models to become standard tools in OS research and therapy development.

## 2 3D OS Spheroid Models

Although this review primarily focuses on scaffold-based OS models, spheroid systems are discussed here to provide a conceptual bridge and comparative understanding of 3D modeling technologies. 3D spheroid models are self-aggregating 3D cell clusters formed in a medium devoid of scaffolds. In addition to improving clinical responsiveness to chemotherapy and advancing personalized cancer care, OS spheroids can be used as a model to study the synergistic effects of cell–cell and cell–matrix interactions.^17^ Scaffold-free spheroids represent the earliest and most accessible format of 3D tumor culture, establishing the foundational knowledge for subsequent scaffold-based advancements. Their inclusion allows for highlighting how key features such as cell aggregation, hypoxic gradients, and limited ECM mimicry influence therapeutic response, thereby emphasizing why scaffold-based systems have evolved to address these limitations.

Organoids, while transformative in epithelial cancer research, were not specifically addressed in this review due to several limitations in their current application to OS. Osteosarcoma, as a mesenchymal tumor, poses distinct technical challenges for organoid generation compared to epithelial tumors. Patient-derived OS organoids remain in the early stages of development and often suffer from issues such as limited proliferation capacity, lack of standardized culture protocols, and difficulties in recapitulating tumor heterogeneity and extracellular matrix architecture.^18^ Due to these technical and biological constraints, organoids have yet to be widely adopted or standardized in OS preclinical research, whereas spheroid and scaffold-based models have more established protocols and demonstrated practical translational utility. Thus, spheroids are presented here as the primary scaffold-free 3D model, laying the groundwork for understanding scaffold-based system advantages.

OS spheroids are routinely generated *in vitro* under nonadherent conditions using ultralow attachment or agarose-coated plates to prevent cell adhesion and drive the self-assembly of cells into three-dimensional aggregates. The culture medium is typically serum-free or contains low serum supplemented with key growth factors such as epidermal growth factor (EGF) and basic fibroblast growth factor (bFGF), which together promote cellular viability and proliferation. Several established methods facilitate spheroid formation, including the liquid overlay technique (using hydrophilic or inert-coated culture surfaces),^19^ the hanging drop approach (where spheroid assembly occurs via gravitational aggregation of cells in suspended droplets),^20^ magnetic levitation systems (where nanoparticles and magnetic fields induce cellular clustering),^21^ and rotary cell culture bioreactors, which simulate microgravity and dynamic cellular interactions;^22^ critically, these methodological choices impact spheroid size, uniformity, and oxygen or nutrient gradient formation—biophysical features that collectively contribute to the spheroids’ biological relevance for modeling the OS tumor microenvironment and stem-like cell behaviors *in vitro* There are several methods of producing spheroids, including the liquid overlay method, hanging drop technique, magnetic levitation facilitated by nanoparticles, and the rotary cell culture method using bioreactors (Figure 1). The choice of method influences spheroid size, uniformity, cellular organization, and the establishment of gradients for oxygen and nutrients—key parameters that impact the tumor-like microenvironment

**Figure 1:**
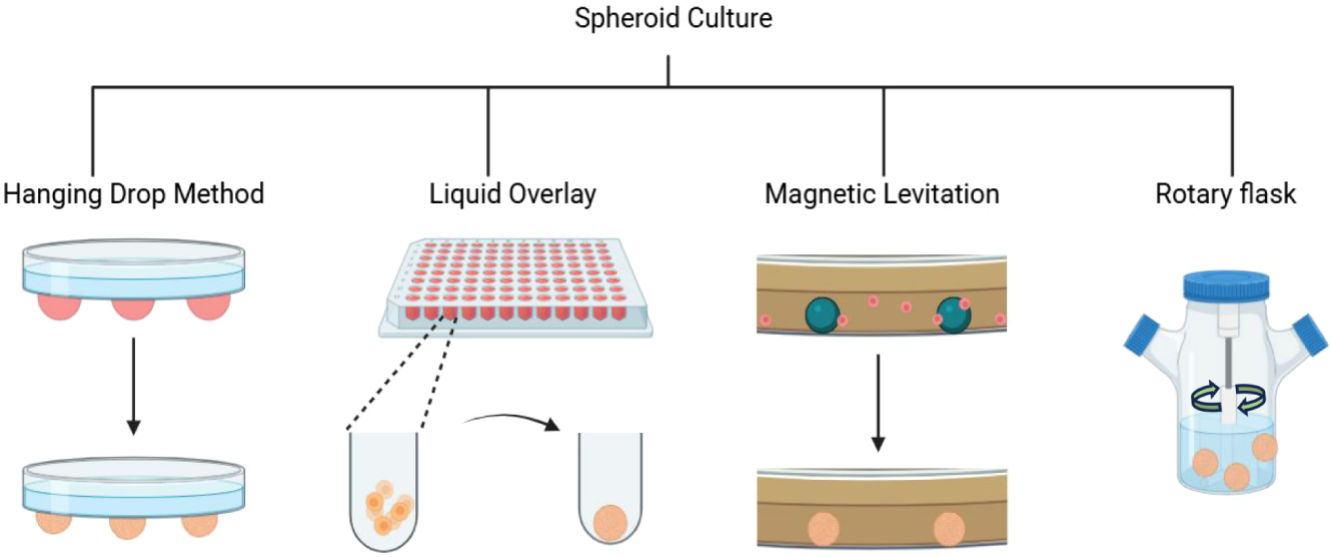
Schematic representation of various methods of self-generating scaffold-free OS spheroids. Figure inspired by^29^ and created using BioRender.

Scaffold-free spheroid models, though not fully replicating the complexities of the *in vivo* TME, are still valuable for osteosarcoma drug screening due to their ability to mimic tumor heterogeneity and facilitate cell–cell interactions. For instance, Ohya et al., demonstrated that MG-63 OS spheroids cultured under serum-free, nonadhesive conditions could be used to evaluate the effect of KCa1.1 channel inhibition, which enhanced the sensitivity of the spheroids to standard chemotherapeutic drugs such as paclitaxel, doxorubicin, and cisplatin.^23^ Similarly, research by Ozturk et al., showed that scaffold-free spheroids derived from Soas-2 osteosarcoma stem cells preserved stem-like properties longer than cells in monolayer culture, making them a more relevant platform for assessing drug responses, particularly against cancer stem cell populations.^24^ Sant et al., conducted a study for generating uniform 3D tumor spheroids using ultralow attachment microplates or polyethylene glycol dimethacrylate hydrogel microwell arrays for cancer drug discovery. The findings revealed that uniform, size-controlled 3D spheroids closely resemble the structural complexity and microenvironment of actual tumors, leading to more physiologically relevant drug response data compared to traditional 2D cell cultures.^25^

TME is extremely complex, and a single tumor spheroid models may not be able to completely mimic the structural complexities.^26^ In order to address this limitation, hybrid spheroid models have been developed by coculturing OS cells along with stromal cells. Spheroids enriched with cancer stem cell (CSC) in OS promote anchorage-independent growth under serum-free, nonadherent culture conditions supplemented with growth factors such as EGF and bFGF for maintaining stem cell phenotype and display tumor-like characteristics *in vitro* and demonstrate tumorigenic capacity *in vivo*.^27,28^ In addition, CSCs contribute to tumor growth, dormancy, metastasis, and recurrence. Therefore, a 3D environment constructed using CSCs and OS cells has been found to serve as a reliable platform for drug screening.^24^ Similar study was conducted by Cortini et al. to generate 3D OS spheroids that mimicked both oncogenesis and the proliferative processes of cells with ECM interactions. This OS spheroid model demonstrated that mesenchymal stem cells (MSCs) and ECM components such as fibronectin, Type I and Type III collagen act as modulators of OS aggressiveness, suggesting the importance of ECM in evaluating drug response against doxorubicin (DOX). Therefore, scaffold-based OS provides more relevant platform compared to spheroids, despite spheroids being considered the gold standard for 3D model.

## 3 Scaffold-Based 3D OS Culture Models

Several types of cells, such as osteoclasts, osteoblasts, endothelial cells, and immune cells, make up the bone microenvironment. The intrinsic complexity of the bone microenvironment is due to a dense mineralized matrix, dynamic mechanical forces, and diverse cell types. The scaffold-based 3D models enable researchers to investigate how physical and biochemical signals shape tumor progression, metastasis, and drug resistance in this microenvironment.^30^ In a study conducted by Yao et al., it was found that scaffolds containing collagen or hydroxyapatite are osteoconductive and osteoinductive, thus increasing OS differentiation and invasion patterns.^31^

In addition to mimicking the ECM, scaffold-dependent 3D models can provide physical reinforcement for the growth of cells. Properties such as stiffness, porosity, and surface chemistry of the scaffolds can be modulated, thereby enabling OS cells to interact with tumor cells and the mineralized matrix. Due to enhanced cell–ECM interactions and altered proliferation rates, OS cells cultured in 3D scaffold often exhibit increased resistance to chemotherapeutic agents compared to their 2D counterparts.^32^ Additionally, scaffold-based 3D models facilitate the incorporation of additional microenvironmental elements, such as stromal or immune cell cultures, hypoxic simulations, and integration with perfusion systems to mimic vascularization. With these advances, complex phenomena, such as immune evasion, angiogenesis, and metastatic dissemination, can be studied, which are vital to OS biology but difficult to study using traditional models.^33^ By including patient-derived cells, these systems become more translationally relevant, paving the way for personalized drug screening and the identification of therapeutic response biomarkers. There are several factors to consider when selecting 3D biomaterials, including biocompatibility, biodegradability, surface attachment, bioactivity, longevity, and the ability to transport oxygen, nutrients, and soluble factors such as growth factors, and drugs.^12^ Various polymer-based scaffolds (natural, native, and synthetic) have been developed for their applications in 3D OS cell culture models (Table 1).

**Table 1:** Overview of Various Natural and Synthetic Scaffold Materials Used in 3D Osteosarcoma (OS) Research, Including Associated Cell Lines and Their Specific Applications in Modeling Tumor Growth, Metastasis, Drug Resistance, and Cell–Matrix Interactions

Scaffold type	Material	Cell lines	Applications in 3D OS research	References
Natural	Matrigel	MG-63, 3AB-OS	Matrigel may act as a signal to induce proliferation and differentiation of 3AB-OS cells inducing tumor growth	^36^
	Silk	SaOS2, HOS	Porous silk sponges support OS cell culture; better mimic angiogenic factor expression and *in vivo* tumor environment	^37^
	GelMA	HOS, 143B, and U2-OS	GelMA/HAMA hydrogels support OS spheroid growth and study of cell–ECM interactions; suitable for bioprinting	^38^
	Alginate	LM8, MG63	Alginate beads encapsulate OS cells for 3D spheroid formation and metastatic studies; higher drug resistance observed	^39^
		OS cells from patient	Single-cell alginate cells expressing cancer stem cell genes (OCT3/4 and Nanog) were responsible conferring resistance to Epirubicin, anticancer drug	^40^
	Methylcellulose	HOS	Used as a hydrogel scaffold for 3D culture; supports OS cell proliferation and demonstrated that the expression of ECM proteins genes were higher in 3D culture compared to 2D	^41^
	Chitosan	MG-63	Chitosan nanofibrous scaffold promoted OS cell attachment, proliferation and osteogenic marker expression	^42^
	Agar	SaOS-2	CSC OS cells incubated in agar gels retained stem cell phenotype for a longer duration in 3D cultures compared to 2D due to higher mRNA expression of *Sox2, OCT*3/4,*Nanog,* and *Nestin*	^24^
	Bacterial cellulose	SaOs-2	Under hypoxic conditions, 3D cancer stem cells in bacterial cellulose scaffold exhibit conservation of phenotype.	^43^
Native scaffold	Collagen	MG-63	Collagen type 1 and hydroxyapatite nanoparticle scaffold demonstrated that 3D environment both protects cells from cold atmospheric plasma (CAP) induced RONS and promotes the stemness phenotype of osteosarcoma cells	^44^
		MG-63, KHOS	Collagen type I scaffolds enhance OS migration and MMP-2/9 expression; useful for metastasis studies.	^45^
		OS LM8	Highly metastatic OS cells in 3D culture had higher proliferative capacity along with secretion of high levels of vascular endothelial growth factor (VEGF)	^46^
		K8	3D collagen sponges promote OS cell proliferation and biosynthesis	^47^
		U2OS	Cells grown in 3D Collagen type 1 scaffold showed reduced proliferation and P13K signaling	^48^
	Hyaluronic acid (HA)	HOS, 143B, and U2-OS	GelMA/HAMA scaffold demonstrated excellent biocompatibility and the OS cells were more sensitive to autophagy directed therapeutics	^38^
	Tricalcium phosphate (TCP)	SaOS2	β-TCP scaffolds enable study of OS invasion and chemoresistance in bone-like microenvironments.	^49^
	Gelatin	SaOS-2	3D bioprinting of sodium alginate hydrogel stabilized by gelatin resulted in marked increase in cell proliferation due to enhanced mineralization in cells	^50^
Synthetic scaffold	PEGDA	MCF7 and MDA-MB-231	PEGDA hydrogel matrix stiffness affects growth and marker expression of CSCs	^51^
	Poly-HEMA	MNNG/HOS	OS cells cultures in Poly-HEMA coated plates had the potential for self-renewal and maintain its potency as they expressed *Oct4* and *Nanog*	^52^
		MNNG/HOS and MG-63	OS cells had the ability to dorm spherical colonies	^53^
	PDLLA	MG-63	Ordered porosity and microstructure of PDLLA scaffold served as excellent substrate for OS cell attachment, growth and proliferation	^54^

### 3.1 Hydrogel-Based Scaffold: Hybrid/Composite Scaffold

Hydrogels are 3D networks of hydrophilic polymers resembling ECM and capable of absorbing thousand times their dry weight without losing structural integrity.^55^ They are valued for their ability to provide soft hydrated environment for cell growth, which is attributed to their high affinity for water content, low antigenicity, tunable mechanical properties, biodegradability, and biocompatibility. These properties facilitate the encapsulation and release of chemotherapeutic agents while stimulating cell proliferation and differentiation.^56^ Cell attachment, migration, nutrient diffusion, and changes in cell behavior should all be possible within an ideal hydrogel scaffold.^57^ Furthermore, hydrogels also serve as a drug delivery platform. In several studies, hydrogels, due to their porous nature, have been demonstrated to be useful for treating tumors, as their biocompatibility and porous structure enable localized treatments.^58–61^

Hydrogels are usually formed using various natural and synthetic components. The biocompatibility of collagen, gelatin, alginate, and chitosan make them good candidates for the development of drugs and cell-based therapies due to their capacity to degrade in the body after the release of drugs or cells.^62,63^ Unfortunately, the lack of durability and mechanical properties limits their application.^64^ Hydrogels are commonly prepared using collagen biomaterial, the most abundant animal protein in the ECM. Gelatin, an alkaline/acidic derivative of collagen, is widely used in tissue engineering hydrogels as it preserves key bioactive cell-binding features, such as RGD motifs, along with metalloproteinase degradation sites.^65^ It also has low cytotoxicity, minimal immunogenic response, and easy modification properties.^66^ Hydrogels can also be prepared using synthetic polymers such as polyglycolide (PGA), polylactide (PLA), polylactide-*co*-glycolide (PLGA), polycaprolactone (PCL), polyacrylamide (PAM), and poly­(D,L-lactic acid) (PDLLA) and do not elicit a body immune response or expose cells to toxicity.^63^ The highly cross-linked 3D networks of hydrogels make them efficient drug carriers, enabling localized delivery and responding to external and internal stimuli. Due to its effectiveness in treating localized conditions, this targeted approach in drug delivery has gained considerable attention in recent years.^67^ Gelatin is one of the most widely used biomaterials for hydrogel preparation since it has the ability to release reactive oxygen and nitrogen species (RONS), when treated with cold atmospheric plasma is beneficial to destroy cancer cells. A 72-hr study conducted by Hsu et al., showed that gelatin-released RONS reduced OS cell survival to 12%–23%. Furthermore, gelatin inhibits MMP-2 and MMP-9 around tumors, hence reducing tumor growth.^68^ Gelatin methacryloyl (GelMA) and Matrigel are the two commonly used natural-based biomaterials for mimicking ECM scaffolds. A study conducted by Monterio et al., investigated the maturation dynamics of MG-63 OS spheroids encapsulated in 10% GelMA and Matrigel hydrogels.^69^ Their study revealed that 3D spheroid cultures in both the hydrogel systems demonstrated increased invasive potential and heightened responsiveness to Lorlatinib (potent ALK/ROS1 inhibitor). Results indicated that cells in 10% GelMA and Matrigel hydrogels were more sensitive to lorlatinib than scaffold-free and scaffold-based 3D spheroid models. By day 14 of culture, spheroids exhibited extensive infiltration into the surrounding hydrogel matrices, mimicking histological and behavioral hallmarks of late-stage *in vivo* tumor progression, thereby not depriving cancer cells of priming factors required for resistance to drugs and cell invasion (Figure 2). The authors highlighted that the cell- laden hydrogels can be used for recapitulating the early stage of OS, while spheroid-based hydrogel platforms can replicate advanced TME and improve preclinical evaluation of targeted therapies.^68^ In a similar study carried out by Peng et al., silk was used as the natural biomaterial for developing biodegradable and injectable silk hydrogels, which were combined with PEG and iodine. It was demonstrated that iodine significantly induces apoptosis in MG63 and Soas-2 OS cell lines by regulating the apoptosis pathway.^61^

**Figure 2:**
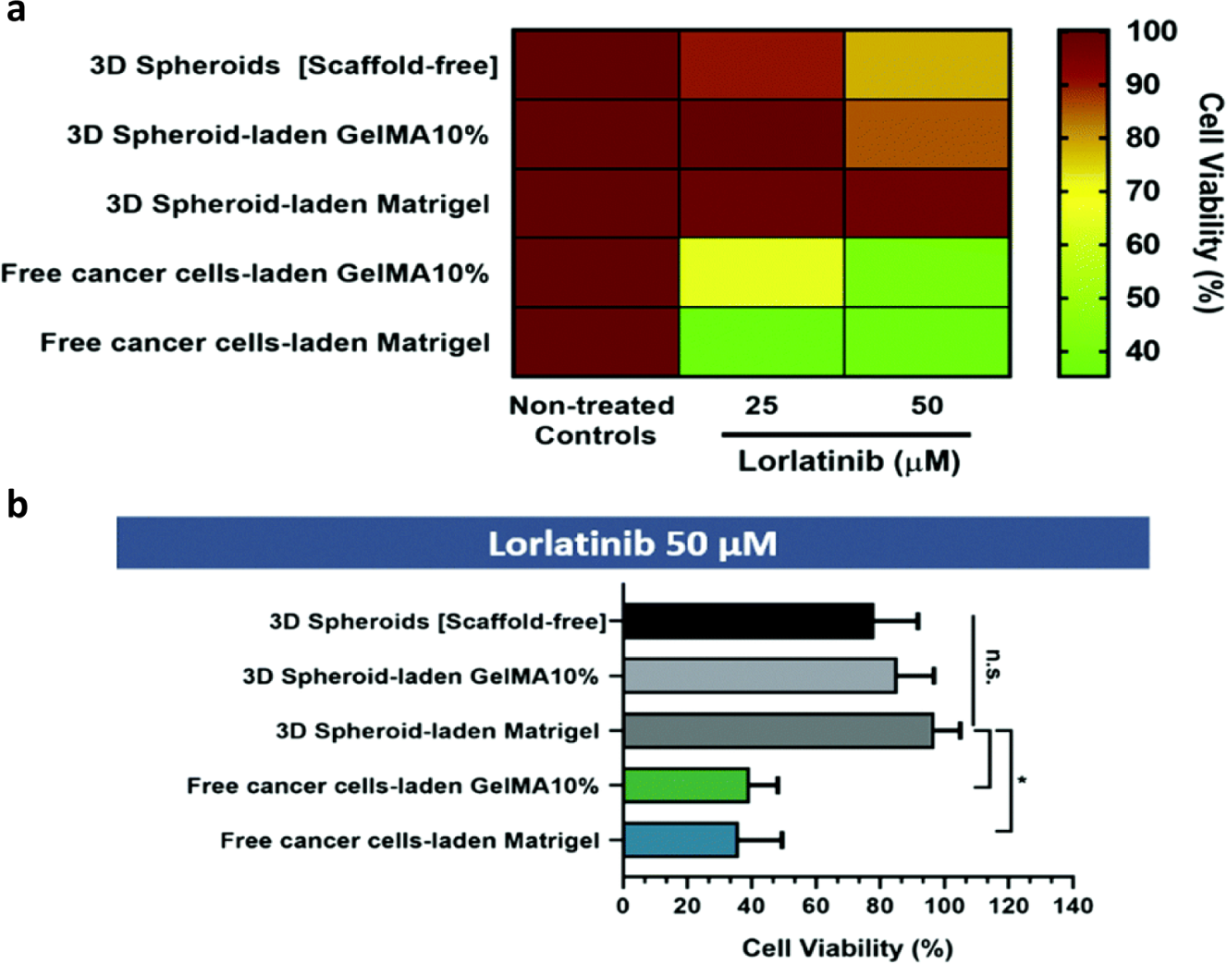
*In vitro* cytotoxicity screening of lorlatinib drug in different 3D OS cultures: (a) Heat map illustrating cell death induced by drug treatment; (b) Quantitative analysis of cell viability in 3D *in vitro* models exposed to the high concentration (50 μM) of lorlatinib. Reproduced from ref 73, under Creative Commons CC BY, cancers (2023).

In addition, integrating hydrogels with other drug delivery systems, such as liposomes or microspheres, enhances their effectiveness of cell or therapeutics delivery.^70^ For example, He et al., photo cross-linked GelMA hydrogel to form a honeycomb-like microspheres and integrated with microfluidics to construct a 3D model for OS. Researchers found greater tumor stemness, proliferation, migration, osteoclastogenetic ability, and resistance to chemotherapy drugs (DOX) in OS cells (K7M2) cultured in 3D GelMA microspheres than in 2D cultures.^71^ Liu et al., prepared dual-network hydrogels by forming a Schiff base linkage between GelMA and oxidized dextran, which is subsequently is subjected to photopolymerization of the methacrylate double bonds, to form methacrylic gelatin/oxidized dextran (GOMP) hydrogel doped with montmorillonitestrontium to deliver DOX using hydroxyapatite (HA) nanoparticles.^72^ It was found that GOMP hydrogel had excellent water absorption capacity, leading to better cell attachment and nutrient delivery. The GOMP hydrogel’s ability to regulate drug release at elevated temperatures and under acidic pH conditions enhances the release of DOX. Consequently, the GOMP hydrogel enables sustained release of the antitumor drug, enhancing the efficacy of localized tumor treatment while minimizing side effects on healthy cells. As hydrogel scaffold applications have advanced, their use has expanded beyond tissue repair to include bone regeneration and tumor eradication in OS (Table 2).

**Table 2:** A comparative summary of recent advances in hydrogel-based platforms for OS therapy, detailing the variety of hydrogel materials, fabrication techniques, therapeutic modalities, and their respective strengths and limitations

Components	Method Used for Hydrogel Culture	Strategies	Advantage	Limitations	References
Chitosan	Ionic gelation, in situ injection	Therapeutic drug delivery	Biocompatible, easy drug loading	Limited mechanical strength, fast degradation	^74^
PEG-Fc doxican	Chemical cross-linking, in situ gelation	Immunotherapy	Prolonged release, tunable propertie	Prolonged release, tunable propertie	^75^
Gelatin methacryloyl	Photopolymerization (UV cross-linking)	Therapeutic drug delivery synergy therapy	Cell-adhesive, supports cell growt	UV may damage cells, limited mechanical strength	^76^
Injectable thermosensitive hydrogel	Temperature-induced sol–gel transition	Simultaneous encapsulation of CA4 and DTX enables their sequential release	Minimally invasive, controlled release	Burst release risk, temperature sensitivity	^77^
Poly(NIPAM-*co*-AM)/MNPs	Site-specific and stimuli-responsive administration of doxorubicin	Magnetic field-responsive gelation	On-demand release, spatial control	Potential toxicity of nanoparticles, cost	^78^
PEGDA and GelMA	Dual cross-linking (chemical and photo	Regulating the integrin-mediated signaling cascade involved in adherens junction dynamics.	Tunable stiffness, supports tissue engineering	Complex fabrication, potential cytotoxicity	^79^
Smart hydrogel (pH/ROS-responsive)	Self-assembly, in situ cross-linking	On-demand, microenvironment-triggered sequential release	Targeted release, reduced side effects	Complex design, scale-up challenges	^80^
Gelatin/black phosphorus nanocomposite hydrogel	Nanoparticle incorporation, in situ gelation	Photothermal therapy + chemotherapy; bone regeneration postablation	Synergistic therapy, imaging capability	Stability, potential nanotoxicity	^81^
Decellularized ECM hydrogel with BMP-2	Decellularization, enzymatic gelation	Enhanced osteoinduction and bone repair after tumor resection	Biomimetic, promotes tissue integration	Batch variability, immune response risk	^82^
Hydrogel with immune checkpoint inhibitors	Injectable, antibody incorporation	Local immunotherapy, reduced lung metastasis	Localized immune activation, reduced metastasis	Short antibody half-life, immune-related toxicity	^83^
Gold nanoparticle-loaded hydrogel	Nanoparticle dispersion, in situ gelation	imaging-guided photothermal and chemotherapy	Dual therapy, real-time imaging	Cost, long-term safety of nanoparticles	^84^

### 3.2 Macroporous Hydrogel Scaffold

#### 3.2.1 Cryogel Scaffold

Cryogels are macroporous hydrogels with distinctive properties, including biocompatibility, biodegradability, interconnected porosity, and chemical cross-linking, which make them superior to other biomaterials, for biomedical applications. They have an advantage over other biopolymers because they are synthesized through the freeze-thawing method at subzero temperatures, during which part of the solvent remains unfrozen and undergoes a series of reactions to form porogen.^85^ Subsequently, the porogen forms an interconnected, stable, and elastic macroporous structure. Cryogels exhibit distinct advantages over hydrogels, including superior mechanical robustness, convenient storage, user-friendly handling, and efficient sterilization capabilities.^86,87^

Hixon et al., fabricated chitosan-gelatin cryogels that were used as transport vehicles for doxycycline-lentiviral transduction of bone morphogenetic protein-2 (BMP-2+), an osteoinductive growth factor, to a permanently defective site for bone regeneration. *In vitro* analysis demonstrated that the bioactive- cryogel scaffold supports bone mineralization, leading to osteogenesis.^88^ In a study carried out by Shalumon et al., gelatin/nanohydroxyapatite cryogels were cross-linked with (1-ethyl-3-(3-(dimethylamino)­propyl) carbodiimide (EDC) or glutaraldehyde (GA), demonstrating that EDC-cross-linked scaffolds favored osteogenic differentiation of bone marrow mesenchymal stem cells (BMSCs) by balancing degradation rates and mechanical stability, while GA-cross-linked scaffolds stimulated cell proliferation.^89^ EDC-nHAP cryogels were successfully used to repair critical-sized cranial bone defects in rabbits, thanks to dynamic bioreactor cultures with cyclic compression, which further optimized osteogenesis and proliferation.

Chen et al., fabricated a cryogel loaded with MXene (Ti3C2) that was able to ablate OS cells under near-infrared (NIR) irradiation while also releasing Sr/Cu/Si ions to facilitate the formation of new bones.^90^ In another study, Shakya *et* al. used polydopamine (PDA)-modified cryogels to enhance chemo-photothermal synergy, resulting in the eradication of tumors in nude mice and the promotion of angiogenesis.^91^ Although there are currently limited studies that explicitly use cryogels for OS treatment, as most research focuses on using cryogels for bone regeneration to repair bone defects, this platform exhibits potential for OS treatment through multimodal therapeutic integration.

#### 3.2.2 Microsphere Scaffolds

The fabrication of microsphere scaffolds ensures consistent pore size, enhances pore connectivity, and maximizes surface area, thereby enabling maximum and controlled delivery of drug molecules.^8^ He et al., developed honeycomb-like porous GelMA hydrogel microspheres using a photo cross-linking technique. These microspheres served as 3D scaffolds designed specifically for the cultivation of OS cells, to provide a biomimetic microenvironment conducive for cell proliferation and interaction (Figure 3). The results demonstrated that these 3D microspheres closely mimic the TME, helping OS cells to retain their natural characteristics and tumor-forming ability. This approach offers a promising new tool for personalized medicine and drug testing in OS research.^71^ The use of microsphere-loaded scaffolds for therapeutic applications facilitates sustained and localized release of chemotherapeutic agents, reducing systemic toxicity and enhancing drug concentrations at tumor sites. Studies have shown that anticancer drug-eluted microspheres, like those containing 5-fluorouracil, paclitaxel, or cisplatin, can inhibit OS cell migration and induce apoptosis.^92^ Cheng et al., demonstrated adriamycin (ADM) loaded gelatin and poly α-lactide-*co*-glycolide (PLGA) microspheres that is anchored to a decellularized periosteum scaffold and had the ability to sustain the release of cancer drugs and suppress cancer cell growth.^93^ This platform enables high-throughput drug screening and localized therapies that aim to eradicate tumors and repair bone defects. Tan et al., developed a hybrid system containing curcumin-microsphere/IR820 (new indocyanine green infrared dye) coloaded methylcellulose hydrogel composites for OS therapy. They demonstrated the injectable curcumin-microsphere/IR820 hybrid hydrogel enabled simultaneous OS eradication through localized photothermal-chemotherapy and subsequent bone reconstruction via sustained curcumin release.^94^ In another study, conducted by Cao et al., encapsulated collagenase (Col) and PLGA microspheres (Mps) carrying Pioglitazone (Pio) and Doxorubicin (DOX) was investigated for OS drug delivery.^95^ The results demonstrated that this method not only achieved robust inhibition of tumor growth and lung metastasis but also minimized toxicity. These findings highlight the potential of such composite systems to enhance therapeutic efficacy against OS by simultaneously addressing tumor proliferation and chemoresistance.

**Figure 3:**
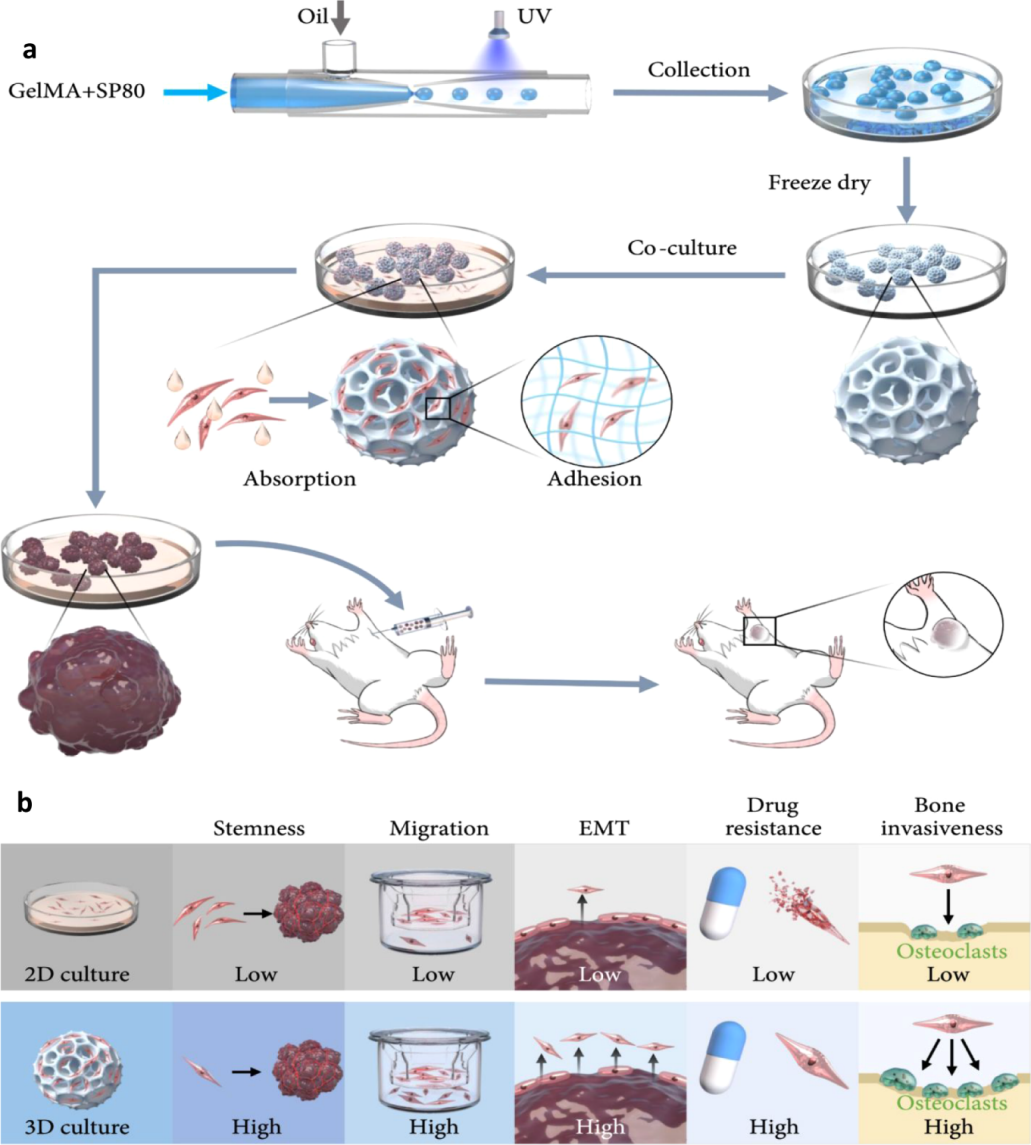
Schematic illustration. (**a**) Fabrication of honeycomb-structured microspheres and their utilization in the development of tumor models. (**b**) Comparison of 3D and 2D cultures of tumor cells based on honeycombed microspheres. Reproduced from ref. 71 under Creative Commons Attribution License (CC BY 4.0), Science Partner Journals, copyright (2022).

### 3.3 Decellularized Scaffold

Decellularized bone scaffolds, in particular, offer a distinct advantage of biomimicking the native ECM composition, which facilitates a more patient-specific response to anticancer therapies.^96,97^ As a result, decellularized ECM offers a physiologically relevant environment for constructing *in vitro* disease models, facilitating more accurate drug response evaluations.^98^ OS signaling and drug resistance profiles were found to be maintained in heterotopic tumors and patient tissues when bone mineral was present in the scaffold.^16^ Unlike soft tissue tumors, OS grows within a more rigid extracellular matrix composed mostly of minerals, such as HA and collagen.^100^ In a recent study conducted by Ren et al., they constructed a novel demineralized bone matrix scaffold (dBMS) obtained from the porcine femur head by decellularization and decalcification. Demineralized bone matrix scaffolds have excellent biocompatibility since they maintain the inherent composition of the natural bone matrix, including HA and type I collagen, and facilitate the proliferation of OS cells, enabling them to form organoids within the porous structure (Figure 4a).^101^ SEM examination revealed that dBMS had a porous structure coupled with a good interconnectivity (Figure 4b). In this study, it was demonstrated that demineralized bone matrix can serve as a potential tool for screening new, effective chemotherapy treatments for OS, as it offers a specific microenvironment within which OS cells are able to persist and develop resistance to drugs such as DOX, similar to the *in vivo* response (Figure 4c,d). A study conducted by Khazaei et al., used pepsin to decellularize the placenta and form hydrogel scaffolds to evaluate the osteogenic properties of SAOS-2 OS cells. It was found that the scaffolds possessed a highly porous, interconnected structure and demonstrated suitable swelling and degradation characteristics, which supported the attachment and proliferation of SAOS-2 cells.^102^

**Figure 4:**
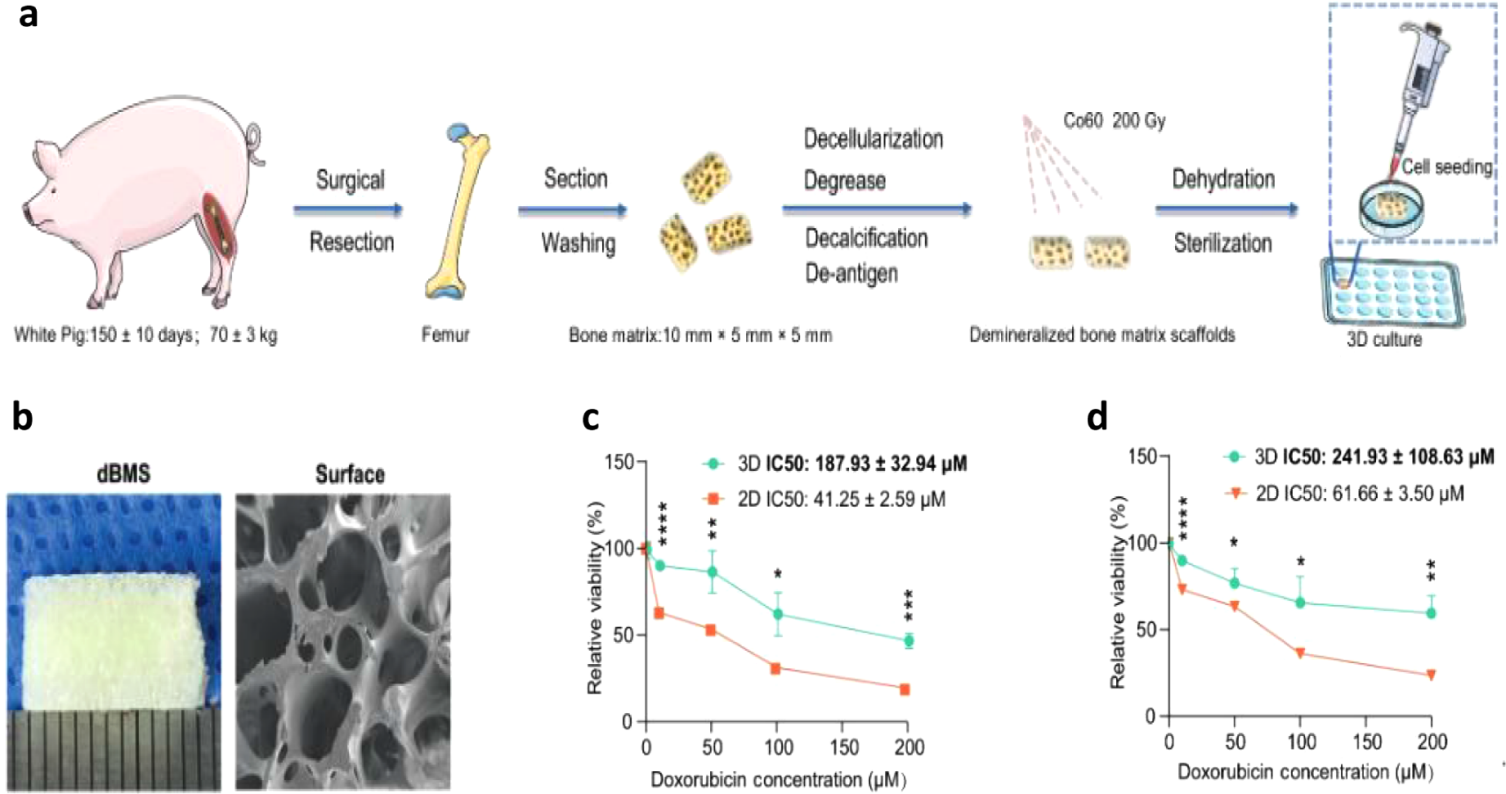
Properties of decalcified bone matrix scaffolds (dBMS): (**a**) A schematic representation of the procedure for preparing dBMS. (**b**) Macroscopic appearance of dBMS and scanning electron micrographs showing cross-sectional views of dBMS. (c,d) (c) Viability of MG-63 (**c**) and MNNG/HOS Cl (**d**) cells treated for 48 h with varying DOX concentrations in 2D and dBMS (3D). Reproduced from ref. 16 under Creative Commons CC BY, International Journal for Cancer, copyright (2024).

In a similar study conducted by Chen et al., which focused on the development of adriamycin-loaded gelatin microspheres incorporated into a decellularized periosteum scaffold obtained through physical and chemical decellularization, the study revealed that the decellularized scaffold provided a biocompatible and structurally supportive environment for the controlled release of adriamycin, resulting in sustained cytotoxic effects against human osteosarcoma cells *in vitro*.^93^ Therefore, through decellularized bone scaffolds, high concentrations of drugs can be delivered to the site of action, thereby reducing systemic absorption and toxicity, while maintaining the space necessary for bone formation.^103^

### 3.4 Microfluidic Chips: Osteosarcoma-On-a-Chip

Microfluidic chips are used to create 3D OS models that more accurately replicate the structure and function of *in vivo* tissue than conventional 2D cultures.^104^ By enabling efficient exchange of nutrients and waste products, this dynamic culture system closely mimics the native TME and allows real-time monitoring of cellular responses to therapeutic interventions. Jaiswal et al., developed osteosarcoma-on-a chip (OOC) model using dual extrusion-based 3D bioprinting technology. The OOC model recapitulated the complexity and spatial organization of cellular and structural components of the TME by using microfluidic bioreactor to mechanically stimulate the cells.^105^ The integration of triculture system, including the tumor and stromal cells along with the microfluidic perfusion enables to explicitly mimic the dynamic *in vivo* physiomimetic conditions, thus it will allow better evaluation and interpretation of anticancer drugs’ efficacy.^106–108^ Preclinical OS drug testing has advanced significantly with the development of OOC, which provides a more accurate, human-relevant, and scalable platform for anticancer drug development compared to static cultures.^109^

Likewise, Lu et al., conducted a similar study on OOC by integrating OS cells in microfluidic device to construct intricate porous microstructures to enable cell–cell and cell–matrix interaction to replicate the *in vivo* OS TME (Figure 5a). Decellularized OS extracellular matrix along with fibrin was loaded with extracellular vesicles of bone marrow-derived stem cells (BMSC-EVs) was used as the acellular bioink to maintain the biochemical properties of the bone tissue. Activation of the OOC system by CXCL12/CXCR4 signaling is associated with heightened OS aggressiveness and accelerated metastasis, as CXCL12/CXCR4 is essential for restoring proliferative signaling in osteosarcoma cells. Based on immunohistochemistry analysis, OOC cells expressed CXCR4 and CXCL12 at levels comparable to xenograft cells and patient OS cells (Figure 5b). To assess the potential of the OOC system as a platform for drug screening, the responses of the model to DOX and plerixafor were investigated. Preliminary findings from the viability assay indicated that plerixafor exhibited a weaker cytotoxic effect compared to DOX. However, when plerixafor (40 μM) was combined with DOX (0.8 μM), there was a marked enhancement in DOX-induced cytotoxicity against patient-derived OS cells. Specifically, cell viability was reduced from 57.07% ± 3.39% with DOX treatment alone to 31.52% ± 4.23% in the combination treatment group (Figure 5c,d).^110^ Therefore, as a drug screening platform, the OOC system may also be used in the future to provide personalized comprehensive treatment.

**Figure 5:**
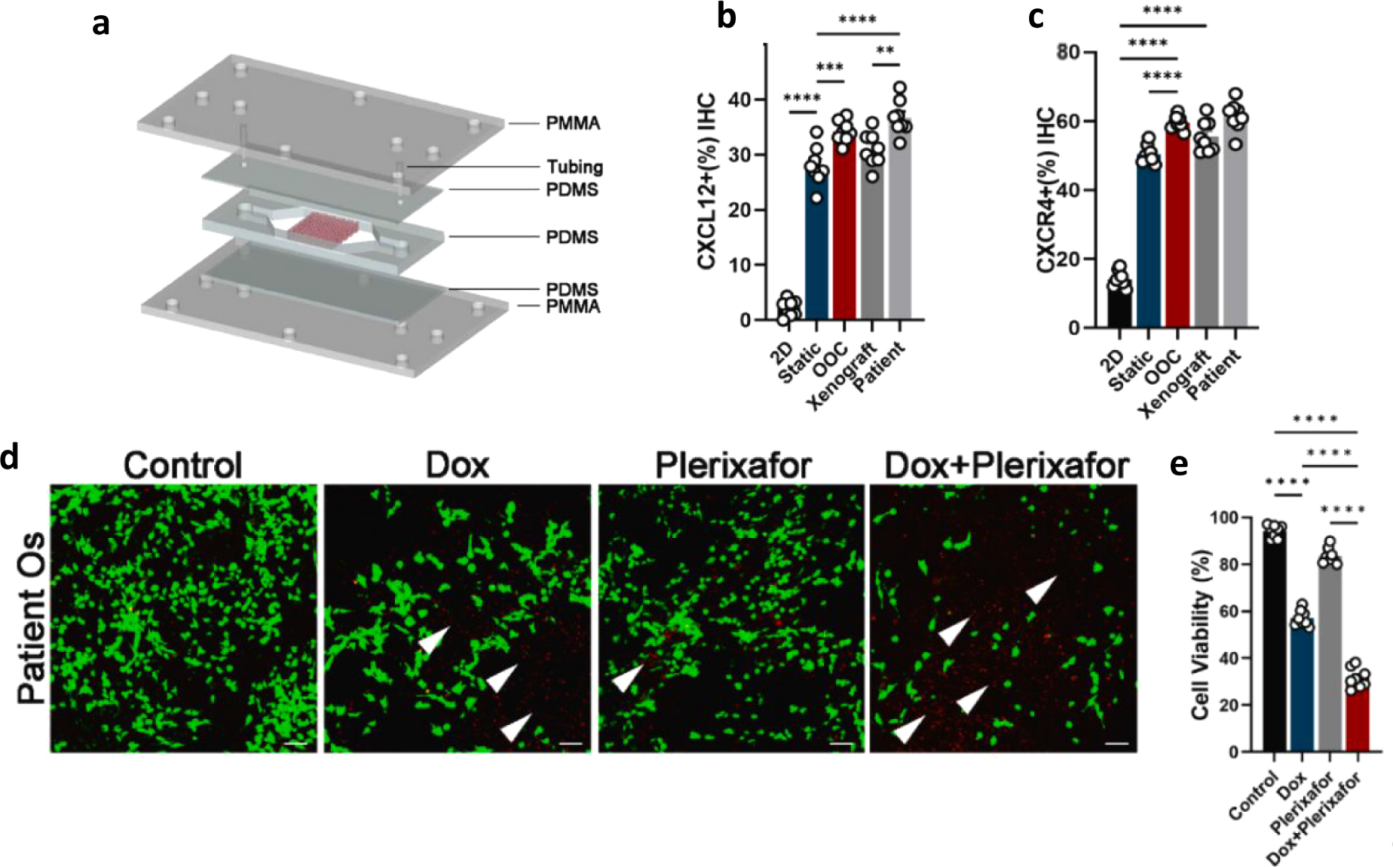
Evaluation of the osteosarcoma-on-a-chip (OOC) system: (**a**) A schematic representation of the OOC model. **(b,c)** Quantitative analysis of (b) CXCL12 and (c) CXCR4 expression levels across 2D culture, static, OOC, mouse xenograft models, and patient-derived OS tissue samples. **(d,e)** Plerixafor combined with DOX drastically enhanced DOX’s killing effect on OS cells. Scale bars represent 50 μm. Reproduced from ref. 110, under Creative Commons CC BY-NC-ND 4.0, Bioactive Materials, copyright (2023).

### 3.5 Nanoparticles/Liposomes-Incorporated Scaffolds

Nanoparticles have the potential of efficiently transporting oxygen, nutrients, and drugs on the scaffold due to its high surface area-to-volume ratio. As a result, nanoparticles are usually combined with the 3D scaffolds for drug delivery applications. Among various nanoparticles, HA nanoparticles (nHA) are the most widely used in OS drug delivery owing to its strong absorption and biocompatibility. Liao et al., developed an innovative bifunctional hybrid hydrogel composed of HA and gold nanorods. OS therapy employed nanohydroxyapatite to promote bone regeneration as it enhanced the bone mineralization capacity at the surgical site when compared to the control group, while the hydrogel matrix allowed them to release and remain localized for prolonged periods of time, which addressed both tumor recurrence and bone defect repair at the same time.^111^

In a study conducted by González Díaz et al., nHA was incorporated into gelatin-based microribbon scaffolds to closely replicate the *in vivo* collagen-mineral matrix of the bone. The study’s findings highlighted HA’s critical role in maintaining OS signaling and influencing drug response by facilitating signal retention and promoting resistance levels akin to those observed in patient-derived tissues and murine tumor models (Figure 6a). Scaffolds containing HA exhibited significantly greater resistance to DOX compared to both 2D cultures and 3D models lacking HA (Figure 6b).^16^ Tornín et al., conducted a similar study by fabricating a 3D model containing nHA and collagen 1 (Col1) to obtain a highly porous, biocompatible, and stable scaffold capable of mimicking the human OS environment. It was found that MG-63 OS cells cultured in Col1/nHA scaffolds expressed increased levels of fibronectin, MMP2, and MMP9. The researchers showed that cold plasma treatment targeted tumorigenicity selectively, and inhibited STAT3 signaling, resulting in reduced tumorigenesis and OS cell viability. It was the first study that demonstrated 3D cultures, when treated with Cold Plasma-Activated Ringer’s solution (PAR) favors the OS cancer stem cell phenotype, resulting in tumor progression.^44^ This study contradicts the previous study conducted by Mateu et al., which demonstrated PAR as a potential therapeutic approach for treating OS.^112^ Wu et al., photo-cross-linked gemcitabine (GEM) hydrochloride-loaded liposomes on GelMA hydrogel to test its efficacy in ablating OS. They developed nanoliposomes using gelatin methacryloyl (GelMA) to investigate their potential for treating osteosarcoma by evaluating their cytotoxic effects on MG-63 cells. By combining gemcitabine hydrochloride with GelMA of an in situ photo-cross-linkable hydrogel, a multifunctional implant with unique antitumor, mechanical, and biodegradable attributes was developed, which demonstrated sustained drug release. Thus, the GEM-loaded lipo-hydrogel approach certainly offers promise for constructing OS implants.^113^

**Figure 6:**
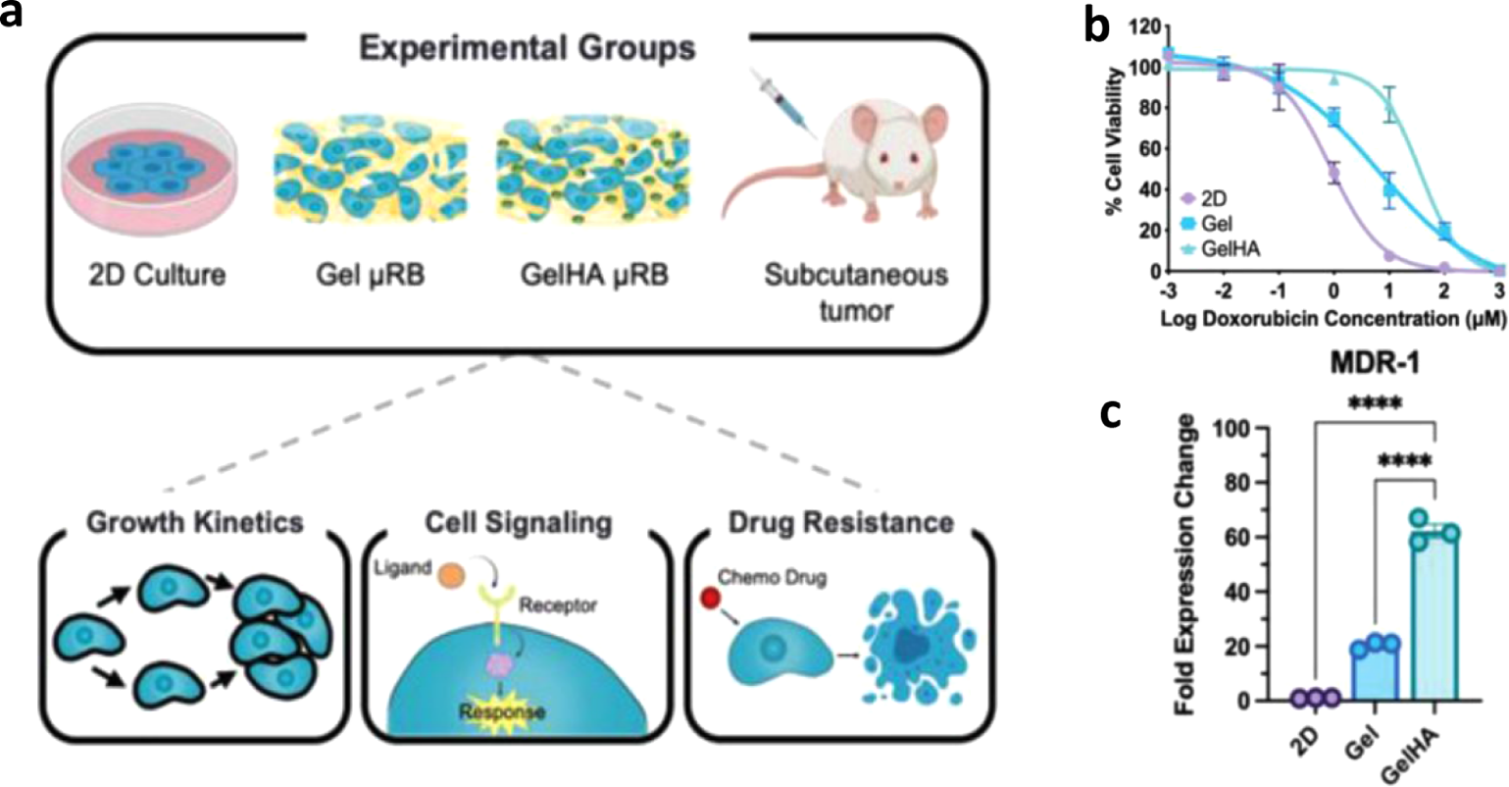
(**a**) Schematic of the experimental setup showing μRB scaffolds with bone-like composition and their effects on osteosarcoma (OS) cell growth and treatment response. (**b**) Dose-dependent viability of OS cells on μRB scaffolds following doxorubicin exposure. (**c**) Quantitative analysis of MDR-1 gene expression in OS cells cultured on μRB scaffolds. Reproduced from ref^16^ under Creative Commons CC BY, Advanced Healthcare Materials, copyright (2022).

## 4 Modeling Osteosarcoma TME

### 4.1 Physiochemical Factors Affecting OS Progression

Biomimetic 3D OS models effectively recapitulates the structural and mechanical properties of bone ECM, which are fundamental to tumor behavior. The use of scaffolds in these models plays a pivotal role, as they influence both mechanical and biochemical signaling pathways that regulate cell–cell and cell–ECM interactions. Furthermore, these scaffolds can simulate the hypoxic and nutrient-deprived conditions typical to the native TME, thereby providing a more physiologically relevant platform for OS research.^114,115^

#### 4.1.1 Mechanical and Biochemical Factors

Scaffold stiffness significantly influences the behavior of osteosarcoma cells, with increased rigidity shown to promote cell proliferation, migration, and resistance to chemotherapy. A study by Lin et al., investigated OS cell behavior within 3D printed GelMA hydrogels of varying stiffness to better understand how mechanical cues influence tumor progression. The key findings revealed that increased matrix stiffness significantly promoted osteosarcoma cell proliferation, invasion, and the expression of genes associated with tumor aggressiveness. Mechanistically, the study identified that stiffer hydrogels activated mechanotransduction pathways, including YAP/TAZ signaling, which contributed to the enhanced malignant phenotype observed.^116^ Furthermore, a comprehensive review by Luu et al., focused on the importance of the physical microenvironment and the activation of mechanotransduction pathways in OS cells like YAP/TAZ.^117^ Another study by Negrini et al., demonstrated that 3D-printed polyurethane scaffolds with adjustable Young’s modulus (0.5–4.0 MPa) provided stable environments for OS cell colonization, with stiffer matrices supporting enhanced tumor cell growth and mimicking the mechanical cues of the bone microenvironment. The optimal pore size and architecture (e.g., 55–67% porosity, interconnected networks) facilitate efficient SAOS-2 cell attachment and proliferation, recapitulating the 3D structure of native bone and supporting more physiologically relevant tumor models.^118^ In addition, scaffold porosity is essential for ensuring adequate nutrient and oxygen exchange, as well as efficient cell infiltration. Miano et al., evaluated an injectable hydrogel composed of porcine bone demineralized and digested extracellular matrix blended with PEGDA, focusing on its suitability for bone regeneration and its interaction with osteosarcoma cells. The findings demonstrated that the hydrogel exhibited a highly porous architecture with interconnected pores, which facilitated efficient nutrient diffusion and cell infiltration. This porous structure was shown to support robust attachment and proliferation of osteosarcoma cells within the scaffold, indicating that the material’s porosity plays a critical role in creating a microenvironment conducive to tumor cell.^119^

The biochemical composition of the scaffold, particularly the inclusion of bone-mimetic minerals like hydroxyapatite (HA), significantly affects OS cell behavior. HA has been shown to promote osteogenic differentiation and provide a favorable environment for OS cell growth, since they support the maintenance of cancer stem cell (CSC) phenotypes and upregulate genes associated with stemness and tumor aggressiveness, such as NOTCH-1 and HIF-1α.^11^ Yao et al., developed bifunctional scaffolds composed of HA, poly­(dopamine), and carboxymethyl chitosan, aiming to combine bone regeneration with antiosteosarcoma properties. The incorporation of HA into the scaffold significantly enhanced osteogenic differentiation and mineralization of bone-forming cells, while simultaneously inhibiting the proliferation of osteosarcoma cells.^120^ Therefore, stiffer, HA-enriched, and highly porous scaffolds more closely recapitulate the native bone microenvironment, thereby influencing OS progression, drug resistance, and metastatic potential.

### 4.2 Cellular Factors Affecting OS Progression

In order to mimic the OS TME, apart from ECM, it should also take into account of the various cell types that are present in the *in vivo* bone microenvironment. Cell culture models based on 3D scaffolds have gained considerable attention in the study of complex interactions between cells and ECM due to their capacity to accurately recapitulate the TME.^121,122^ The OS TME is a highly complex microenvironment comprising a diverse array of cell types, such as mesenchymal stem cells and fibroblasts, stromal cells, osteoblasts, osteoclasts, osteocytes, endothelial cells, hematopoietic cells, various immune cells-including lymphocytes and macrophages-and adipocytes, all embedded within a mineralized ECM. Tumor cells interact with multiple bone microenvironment cells to drive osteolytic destruction. MSCs generate osteoblasts and support HSPCs, which give rise to immune cells and osteoclasts. Tumor-derived factors (e.g., VEGF, TGF-β, PGE2) promote angiogenesis, immunosuppression, and osteoclastogenesis. Osteoclast activity releases ECM growth factors, further stimulating tumor growth and sustaining the destructive cycle (Figure 7). Through the crosstalk among these cells, tumor cells can evade immune detection, promote angiogenesis, cell intravasation, dissemination of cancer cells, and dysregulate bone remodeling.^123^

**Figure 7:**
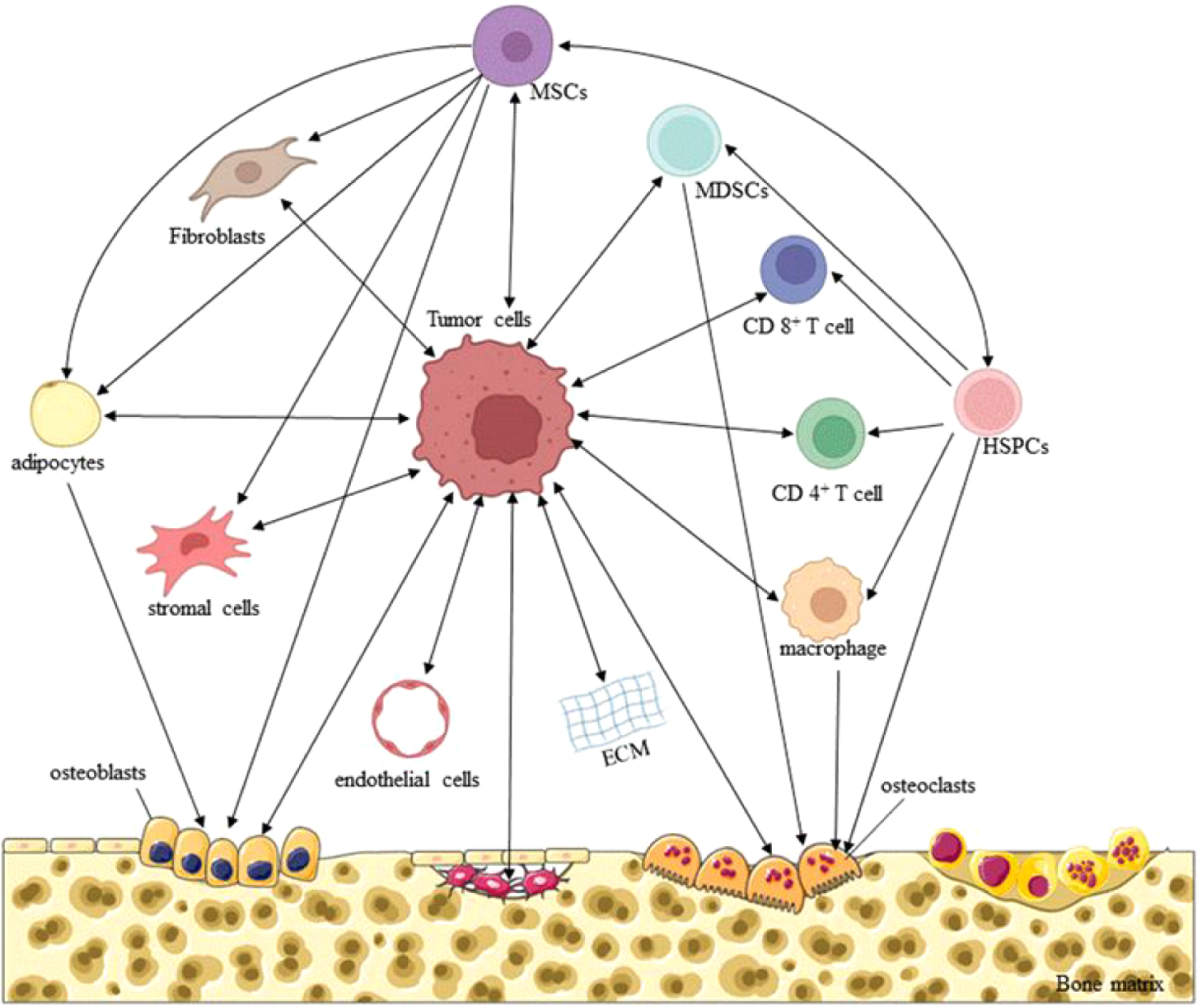
Interactions of tumor cells with the bone microenvironment. Reproduced from ref. 126 under Creative Commons CC BY, cancers, copyright (2022).

Bone marrow-derived mesenchymal stem cells (BMSCs) are a key component of the OS TME, exhibiting a pronounced affinity for OS cells. Upon interaction, BMSCs differentiate into cancer-associated fibroblasts (CAFs) and secrete cytokines such as IL-6, IL-8, and MCP-1 within the TME. These factors collectively promote increased OS invasiveness, motility, and transendothelial migration.^124^ In a study conducted by Costa et al., a fully humanized 3D *in vitro* OS model, recapitulating TME using OS tumor cells, mesenchymal stem cells (MSCs), and immune cells, specifically tumor-associated macrophages (TAMs), to form a multicellular tissue spheroid (MCTS).^125^ It demonstrated that OS 3D models mimicking the TME had higher efficacy in drug screening. Therefore, in order to accurately replicate the *in vivo* physiopathological condition of the tumor, the 3D OS model should be a reliable representation of the ECM with cellular heterogeneity, incorporating various cell types along with the OS cells.^32^

## 5 Drug Resistance Dynamics in Biomimetic OS Models: 2D vs 3D

The high attrition rate of therapeutic agents in clinical trials makes drug discovery and development a time-consuming and expensive process.^127^ For decades, 2D models have served as gold-standard models for high-throughput drug screening and drug toxicity analysis, apart from their non-negotiable role in biomarker discovery and studying disease pathology. However, they have failed to reiterate the major *in vivo* features, leading to their poor translational ability, since they lack tissue specificity, cell–cell, cell–ECM interactions, as well as biochemical and mechanical cues. It is, therefore, evident that these models are weaker when it comes to predicting the efficacy of potential drugs for certain diseases, such as cancer.^12^ When compared to 2D models, 3D systems are the most accurate representations of *in vivo* cellular phenomena. One of the key benefits of using 3D OS models is that they can be used to determine the efficacy and toxicity of therapeutic candidates before drugs enter clinical trials.^128^

Several studies have consistently demonstrated that OS cells cultured within 3D scaffolds can resist chemotherapeutic agents more effectively than cells cultured on 2D surfaces. This is due to the fact that, in 3D cultures, a complex matrix incorporating the OS niche^69^ and native ECM^73,129^ components is responsible for enhancing OS cell resistance to anticancer drugs. Additionally, osteomimetic native materials, such as HA, are associated with higher drug resistance since they have been found to enhance resistance phenotypes, aligning with *in vivo* models and patient-derived tumor responses.^16^ As a result, IC_50_ values of drugs, including methotrexate, doxorubicin, cisplatin (MAP), and non-MAP agents, are usually higher than those observed in 2D monolayer cultures, suggesting that 3D OS models exhibit higher drug resistance (Figure 8). Torin et al., demonstrated that PAR-treated 3D cultures of MG-63 cells on Col1/HA scaffolds had a higher proliferation rate as they induced cancer phenotype stemness, in contrast to the reduction in viability observed in 2D cell cultures.^44^ The 3D OS hydrogel microsphere cultures demonstrated enhanced resistance to the chemotherapeutic agent DOX compared to conventional 2D systems. This increased drug tolerance was supported by molecular analyses showing a 3.2-fold upregulation in BCL-2 gene expression (an antiapoptotic regulator) and an 18.46% reduction in Annexin V-positive cells (indicative of early apoptosis) within the 3D microenvironment. These quantitative differences in apoptotic markers suggest that the spatial architecture of 3D culture systems may promote cell survival mechanisms under chemotherapeutic stress, thereby conferring resistance to the cells.^130^

**Figure 8:**
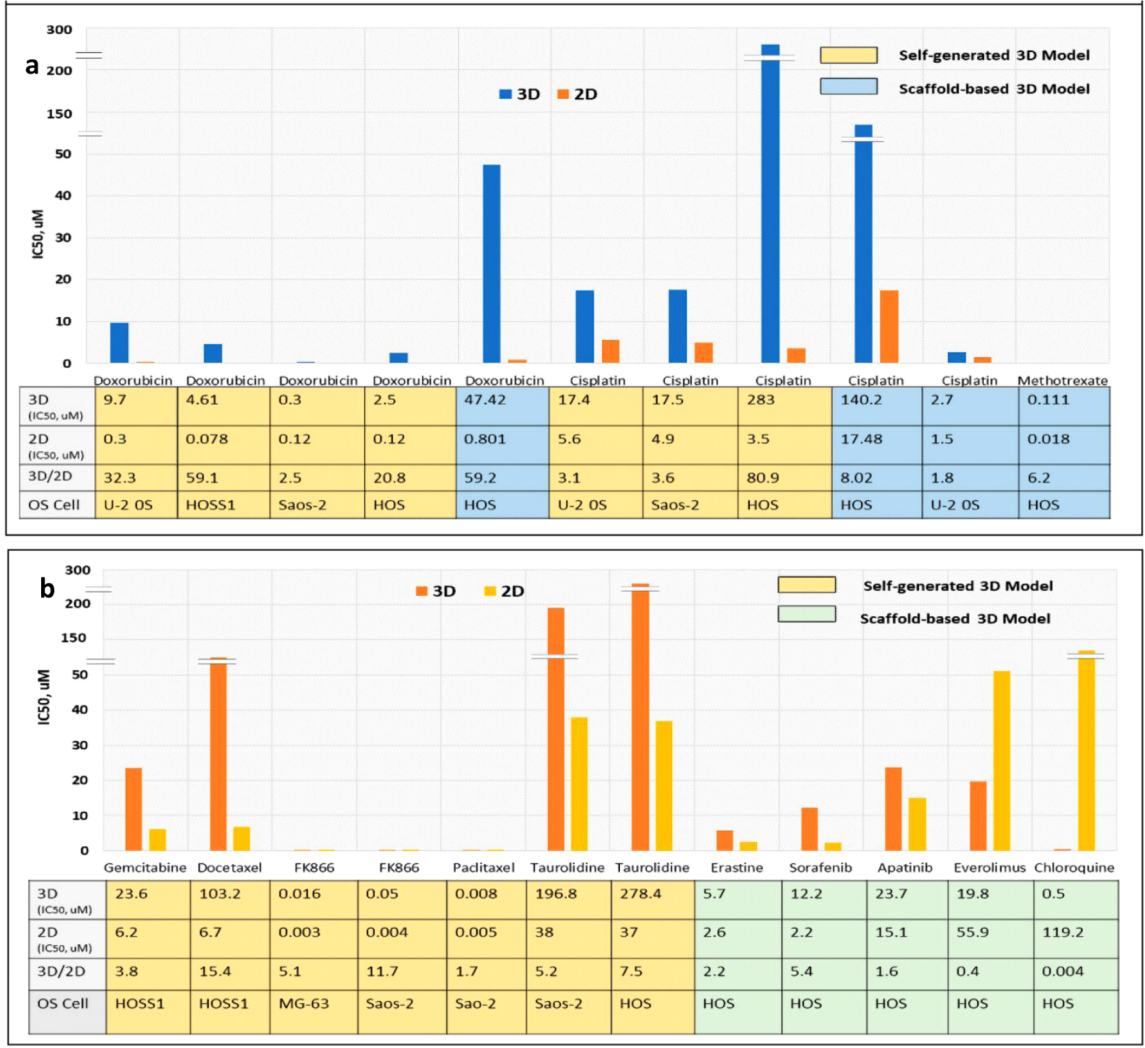
3D OS models for anticancer drug screening. (a) Comparison of IC_50_ values and 3D-2D IC_50_ ratios for doxorubicin, cisplatin, and methotrexate (MAP regimen) drugs. (b) Comparison of IC_50_ values and 3D-2D IC_50_ ratios for non-MAP drugs. Reproduced from ref. 32, under Creative Commons CC BY, cancers, copyright (2023).

In addition to enhancing drug resistance, coculturing OS cells with stromal components within these scaffolds increases osteogenic differentiation, mineralization, and angiogenesis in the ECM. This is attributed to the fact that the stromal cells facilitate the remodeling of the ECM, promote tumor cell migration through the release of various cytokines and chemokines, and stimulate neoangiogenesis.^131^ Therefore, using stromal cells such as osteoblasts and fibroblasts, anticancer therapy has been shown to have selective toxicity toward OS cells while sparing the normal stroma. It was shown by Dobos et al. that irradiation of 3D cultures of adipose-derived stem cells (ASCs) with OS spheres did not cause damage to healthy cells after two-photon excited photodynamic therapy (TPE-PDT), demonstrating the precision of irradiation.^132^ As a result of the transition from 2D to 3D OS cell culture, drug development poses a significant advantage at the later stages of clinical trials. However, 3D OS models are not all advantageous as they are also associated with certain limitations with respect to reproducibility of the results and facilitating high-throughput drug screening.^133^

## 6 Challenges and Future Perspectives

OS is the predominant subtype among sarcomas, accounting for more than 61% of cases. Despite its prevalence, this malignancy is associated with a considerable risk of limb amputation, and the overall five-year survival rate remains poor, typically falling below 20%.^134^ Despite notable advances in scaffold-based 3D cell culture models for OS, several key challenges remain unresolved. The complexity of the bone TME, characterized by a dense mineralized matrix, diverse cell populations, and dynamic biochemical and mechanical cues, is difficult to fully recapitulate *in vitro*, even with advanced 3D scaffold-based systems.^30^ Although natural biomaterials such as collagen and gelatin offer excellent biocompatibility, their limited mechanical strength and durability restrict their long-term application in OS modeling.^62,135^ Conversely, synthetic polymers provide tunable physical properties but can induce cytotoxicity or immune responses, necessitating careful biomaterial selection and optimization.^63^ Standardization and reproducibility also pose significant barriers, as variations in scaffold fabrication, cell seeding density, and culture conditions can lead to inconsistent experimental outcomes.^34,35^ Furthermore, the integration of multiple cell types—such as stromal, immune, and endothelial cells—adds complexity to model design and maintenance, yet is essential for accurately mimicking the TME.^30,33^ From a translational perspective, the scalability of these models for high-throughput drug screening remains limited compared to traditional 2D systems, and correlating *in vitro* drug responses with clinical outcomes is still a major challenge.^8,136^

Looking forward, several promising directions could address these limitations and enhance the translational relevance of scaffold-based 3D OS models. Advances in biomaterials, such as the development of hybrid or composite scaffolds, may offer improved mechanical properties and bioactivity, enabling more accurate simulation of the bone microenvironment.^56^ The incorporation of patient-derived cells and organoid technologies could further increase physiological relevance and support personalized medicine approaches.^16,33^ Emerging biofabrication techniques, such as 3D bioprinting and microfluidic integration, hold potential for creating highly reproducible, customizable models that incorporate multiple cell types and simulate vascularization. In a recent study conducted by Smith et al., they inserted OS cells to the bone core developed from human trabecular bone and implanting them on the chorioallantoic membrane from fertilized chicken to develop vascularized 3D bone model. The authors demonstrated that this 3D model successfully mimics the OS TME including the expression of the markers such as CD68 and CD105. The experimental system enabled assessment antiosteosarcoma effects of mifamurtide drug, which led to decreased tumor-associated biomarkers and enhanced bone volume restoration. These findings highlight the model’s utility as a biologically relevant tool for studying TME dynamics and evaluating therapeutic candidates, offering a robust framework to bridge the gap between experimental studies and clinical applications.^62^

Standardization of protocols, quantitative imaging, and the use of omics-based analytical tools will be critical for improving reproducibility and enabling robust cross-study comparisons.^6^ Ultimately, the convergence of advanced biomaterials, patient-derived systems, and high-content analytics is expected to drive the next generation of scaffold-based 3D models, accelerating the discovery of effective therapies and deepening our understanding of OS biology.

## 7 Conclusion

This review provides a critical overview on the current landscape of scaffold-based 3D cell culture models for OS research and drug screening. Scaffold-based 3D models offer significant advantages over traditional 2D cultures by more faithfully replicating the architectural, mechanical, and biochemical complexity of the OS TME. These models enable more accurate representation of tumor–stroma interactions, drug resistance mechanisms, and cellular responses to therapeutics, thereby bridging the translational gap in predicting preclinical drug testing. Scaffold-based systems, particularly those utilizing biomimetic and tunable materials, provide versatile platforms for both fundamental research and the development of personalized medicine strategies. However, the review also highlights persistent challenges, including the need for improved standardization, scalability, and integration of patient-derived cells to fully harness the potential of these models. Future advancements in biomaterials, biofabrication techniques, and multicellular coculture systems are expected to further enhance the physiological relevance and translational utility of scaffold-based 3D models. In summary, this review underscores the promise of scaffold-based 3D culture systems as transformative tools for OS drug discovery and personalized therapy, while also emphasizing the necessity for continued innovation and rigorous validation to establish these models as standard platforms in preclinical research.
